# Carrying the Facilitator Responsibility: Experiences of Healthcare Professionals Under Training to Become Facilitators of Ethics Case Reflection Rounds in Paediatric Oncology

**DOI:** 10.1007/s10730-025-09572-7

**Published:** 2025-11-25

**Authors:** Cecilia Bartholdson, Bert Molewijk, Margreet Stolper, Pernilla Pergert

**Affiliations:** 1 Childhood Cancer Research Unit, Department of Women’s and Children’s Health, Karolinska Institutet, Stockholm, Sweden; 2 Medical Humanities, Amsterdam UMC, Amsterdam, The Netherlands; 3 Centre for Research Ethics & Bioethics, Department of Public Health and Caring Sciences, Uppsala University, Uppsala, Sweden

**Keywords:** Classic grounded theory, Ethics case reflection, Ethics support, Facilitator, Moral case deliberation, Qualitative, Responsibility

## Abstract

In paediatric oncology, ethical dilemmas often arise and includes different perspectives about what is perceived as ethically right care for the child. Ethics Case Reflection (ECR) rounds, a form of Clinical Ethics Support (CES), offer a structured, dialogical approach to facilitate ethical reflection. A Nordic working group on ethics in pediatric oncology offers a training program for healthcare professionals to become facilitators for ECR rounds. The aim of this study was to explore experiences of these facilitator trainees when facilitating ECR rounds in Nordic paediatric oncology. Using classic grounded theory methodology, data were collected through three focus groups with 22 Nordic facilitator trainees and 27 individual interviews with 17 facilitator trainees from Sweden. *Carrying the facilitator responsibility* is the core category in this study, used to resolve the main concern of delivering a meaningful experience of ethics support and enabling ethically good care for the child. To carry the facilitator responsibility and handle associated challenges, the condition of *achieved facilitator confidence* and the strategies of *allying* and *undertaking the facilitator role* is important. ECR facilitator trainees take their role seriously as they are carrying the facilitator responsibility to deliver a meaningful experience of CES and enable ethically good care for the child. We conclude that this perceived burden of responsibility should be better addressed during future facilitator trainings, emphasising the use of various strategies to decrease this burden and share the responsibility for the ECR together with the ECR participants. Training also needs to strengthen ethical competence to achieve facilitator confidence of the trainees. Further research is needed on what kind of core ethical competencies facilitators of ECR rounds needs.

## Introduction

Healthcare professionals (HCPs) working in paediatric oncology frequently encounter ethical dilemmas involving moral uncertainties in the care of vulnerable children and their families. In these situations, navigating potentially conflicting values, emotional burdens, and complex decisions are often required. In order to learn to handle these ethical dilemmas and promote joint ethical reflection in multidisciplinary teams, structured Clinical Ethics Support (CES) can be helpful. CES has become an established and acknowledged service in many healthcare organisations in Europe. One of the forms of CES is Ethics Case Reflection (ECR) rounds, also referred to as Moral Case Deliberation (MCD) or Ethics Reflection Groups (ERG); synonymous for the same form of CES and independent of the specific conversation method used.

ECR rounds entails a structured methodological approach in a group setting, guided by a trained facilitator or ethicist, using dialogue as a tool to reflect on individuals’ experiences involving an ethical question and focus on joint ethical understanding and learning (Bartholdson et al., 2014). This approach distinguishes from traditional Clinical Ethics Consultation (CEC) or Moral Counselling as it is primarily focused on facilitating a joint moral inquiry and exchanging perspectives from stakeholders involved, to gain both a collective and individual ethical knowledge and create moral wisdom rather than through expert facilitation and guidance (Delany et al., 2024). Various evaluation studies have shown that the dialogical approach of ECR rounds suits many different goals at the same time, such as facilitating a permissive dialogue, foster shared understanding, providing a basis for decision making, strengthening ethical competences, and developing policies (Bartholdson et al., 2016, 2022; Haan et al., 2018; Hem et al., 2015; Weiner et al., 2021). Research have also shown that ECR rounds enables HCPs to deal more constructively with their ethical dilemmas and increase the cooperation in interprofessional teams (Bartholdson et al., 2018, 2022; Haan et al., 2018; Hem et al., 2015; Janssens et al., 2015; Silén & Svantesson, 2019; Weiner et al., 2021).

ECR rounds are guided by a trained facilitator who uses a dialogical approach during the moral inquiry into a moral question in which participants reflect on their ethical dilemma in a case (Stolper et al., 2015). The facilitator should be neutral regarding the content of the case and promote an open and safe space in which participants have an equal say (Metselaar et al., 2015). To guide and support the dialogue and reflection process, the facilitator can use one of the many conversation methods. Examples of conversation methods are the Dilemma method (Stolper et al., 2016), the Karolinska model (Bartholdson et al., 2014), and the SME model (Forde & Pedersen, 2011). Facilitating ECR rounds requires a distinct set of competencies, including ethical sensitivity, dialogical skills, and the ability to guide group reflection without imposing personal views. The facilitating role is non-authoritarian and focuses on facilitating HCPs to deliberate for themselves and come to their own moral conclusions in the case at hand (Pergert et al., 2025). The facilitators, and also the ECR round, have no legal mandate or responsibility to make decisions. Thus, the professional responsibility of the decision remains with the HCPs.

In the past two decades, various training programs have been developed in Europe for HCPs to gain and develop the expertise of a facilitator of ECR rounds (Plantinga et al., 2012; Stolper et al., 2015) or ethics support personnel in general (Dörries et al., 2014). In the Nordic countries, a facilitator training programme was developed and has been offered since 2017 within a Nordic working group on ethics to prepare HCPs to guide ECR rounds in paediatric oncology. The training was developed and offered by members of a special interest group in the Working Group on Ethics of the Nordic Society of Paediatric Haematology and Oncology (NOPHO), and NOPHO Nursing. This training, inspired by established Dutch models (Stolper, 2016), combines theoretical knowledge with practice in clinical settings. For improving facilitator training and ensuring the sustainability and quality of CES in paediatric oncology, it is important to evaluate and gain knowledge about the facilitators experiences of facilitating ECR rounds in their clinical settings.

## Aim

To explore experiences of HCPs, under training to become facilitators, of facilitating ECR rounds in Nordic paediatric oncology.

### Research Question

What is the main concern of HCPs, under training to become facilitators, when facilitating ECR rounds in paediatric oncology, and how do they handle it?

## Methods

In this qualitative study, classic grounded theory methodology was employed to explore how HCPs handled their main concern when facilitating ECR rounds (Glaser, 1998; Glaser & Strauss, 1967).

### The Facilitator Course

The facilitator course was structured into three parts: a three-day introductory part, a two-day follow-up part about six months later, and a practical part after the introduction and after the follow-up. In the practical part participants were expected to conduct ECR rounds in their clinical settings, performing approximately 4–8 rounds as facilitators or co-facilitators. In the first cohort of the course 29 HCPs, recommended by their managers, participated including 21 participants representing all six paediatric oncology centres in Sweden and eight participants from the other Nordic countries.

### Participants and Data Collection

We invited facilitator trainees from the first cohort of the course to participate in this study by taking part in a focus group interviews and in individual interviews. Data were collected by focus group interviews (*n* = 3) in person with trainees from the Nordic countries: Sweden, Denmark, Finland, Iceland, and Norway (*n* = 22). The focus groups consisted of facilitator trainees who were registered nurses, physicians or social workers. These focus groups took place at the beginning of the second part of the course, about six months after the introductory part. The focus group interviews were moderated and observed by the researchers, who were also teachers in the course, and the first author who was not a teacher in the course. When moderating, the researchers used an interview guide to foster interaction and discussion within the group while the observers took field notes (Bloor et al., 2001).

Besides the focus groups, Swedish trainees from all paediatric oncology centres were invited and 17 out of 21 agreed to take part in individual semi-structured telephone interviews. The individual interviews were done, by the first author, three months after the introductory part (*n* = 14) and three months after the follow-up part (*n* = 13) of the course. Examples of questions in the interviews were: How has it been to facilitate ECR rounds in the team? How do you see your role as a facilitator? What strategies do you use? The Swedish trainees participating in the study had an average of 11 years of work experience in paediatric oncology, with a range spanning from 1 to 30 years, and included representation from various professional groups and nationalities. Three trainees declined, two had ended their position and one did not have time. All interviews were sound-recorded and transcribed, and field notes were written.

### Data Analysis

Data collection and analysis were carried out in parallel, according to classic grounded theory (Glaser, 1978, 1998; Glaser & Strauss, 1967). The first author read through fieldnotes and transcribed text to gain a comprehensive understanding of what the trainees expressed in the focus group interviews and individual interviews. Coding of the data included open coding, selective coding and theoretical coding (Glaser, 1978). Open coding, line-by-line, was conducted using a software for qualitative analysis (NVivo). Using grounded theory, it is not uncommon for several core categories to emerge and in that case, the researchers need to focus on one at a time (Glaser & Strauss, 1967). In this study, two core categories emerged. One core category, which is covered in this article, concerned facilitating ECR rounds in the clinic. The other core category concerned the implementation of the ECR rounds (Pergert et al., 2024) and is published separately. In the present study, the first author continued the analysis with selective coding of data pertinent to the core category. Codes and categories were continuously compared and discussed among the authors. Memos were written to document the categories and their properties. Additionally, memos were sorted and theoretical coding was performed manually to integrate the categories (Glaser, 1978).

## Findings

*Carrying the facilitator responsibility* is the core category in this study. It is used to resolve the main concern of delivering a meaningful experience of ethics support and fostering ethically good care for the child. To carry the facilitator responsibility and handle associated challenges the condition of *achieved facilitator confidence* and the strategies of *allying* and *undertaking the facilitator role* is important.

### Carrying the Facilitator Responsibility

Carrying the facilitator responsibility is about feeling a heavy burden of responsibility but also trying to take on the responsibility for *the case*,* the ECR participants* and *the process* as well as adhering to one’s personal standards as facilitator and high demands, as exemplified by this quote:

But it is precisely this that we provide a lot of value, and it turns out that you set very high demands. Maybe you should be a little more relaxed and not make those demands, ‘oh I have to be really, really good’….

In relation to *the case*, carrying the facilitator responsibility entails feeling and taking on great responsibility for identifying the ethical dilemma and what is important followed by relevant actions in the case, as it could influence the care of the child.

In relation to *the ECR participants*, carrying the facilitator responsibility is about providing a good experience and having ethical competence and tools to get to the ethical aspects of the case. When feeling and taking on responsibility for the ECR participants experience, the facilitators perceived challenges. Challenges included that sometimes HCPs were uncertain to give their views and did not know how to formulate what was important for them, mostly depending on the unfamiliar situation. Facilitators in our study explained that when uncertainty about giving their views and how to formulate values occurred, facilitators tried to guide the reflection and encourage the ECR participants to assist each other in formulating different ways of expressing perspectives.

In relation to *the process*, carrying the facilitator responsibility is about being responsive to the latent meaning of what is being expressed and posing questions to encourage participants to think beyond social facades and preconceived notions. It is also about encouraging everyone to feel that they are able to articulate their individual perspective. When feeling and taking on responsibility for the process, participants experienced challenges. Challenges included getting stuck in the ethical analysis of the case without knowing how to proceed and deciding on what ethical question to reflect upon. Furthermore, dealing with HCPs that tend to end up in problem solving and to rush to a conclusion, was also challenging for the facilitators. Moving forward to the next steps within the ECR conversation method when guiding the dialogue was also sometimes hard. Participants in the present study explained that HCPs sometimes get so engaged that they start to talk about things not relevant to the ethical dilemma; thus, showing that the HCPs sometimes struggled to focus on the specific case. Challenges for carrying the facilitator responsibility involved feelings of being insufficient both theoretically and practically.

### Achieved Facilitator Confidence

Achieved facilitator confidence is a condition for carrying the facilitator responsibility and undertaking the role as facilitator. Achieving facilitator confidence involves feeling safe in the role and in the ECR round process and includes safe practicing and developing theoretical knowledge.

*Safe practicing*: Almost all participants expressed a great need for exercise to get extended skills because they experienced practical insufficiency. For example, not feeling able to document the ethical analysis on the flip chart and guiding the dialogue at the same time. Safe practicing was conducted by repeated exercise in a safe group. A safe group was a small group at the clinical ward or within a small group of facilitator trainees only. The examples of small groups were perceived as a safe environment for exercise and mutual learning. As expressed by one participant: “The exercise itself within a small safe group, like, was a key for me”. Another participant expressed:

But I think you need to do it many times to have it in use. Do this more. I think you need some time. And then when maybe a year or so has passed,… or maybe not a year, but when you’ve managed to do 30 [ECR rounds], something like that.

*Developing theoretical knowledge*: Participants described a desire for theoretical education because they experienced theoretical insufficiency including lack of ethical reasoning, leading to challenges identifying the ethical dilemma and what is important followed by relevant actions (values and norms). Some wanted to take a course in ethics including ethical theories and models, others wanted to attend ethics-seminars and workshops. The main reason for the need for achieved facilitator confidence was that many ECR trainees assumed that it would impact their ECR facilitator role as well as the outcome of the ECR. One participant expressed it like this:

But it would be nice for yourself to have more theoretical knowledge in depth, that it might make it easier for you when you try to help people find, the most important thing….maybe it would be good if you yourself had more solid like foundational knowledge in ethics, it would be easier.

### Allying

Allying is a strategy for carrying the facilitator responsibility and is about sharing the responsibility by working together with fellow facilitators and providing mutual support and assistance when facilitating. Allying is also about making efforts to achieve the shared goal. Facilitators are supporting each other in guiding the group and sometimes share the role as facilitators or having co-facilitator roles.

Participants in our study expressed that they were energized by fellow facilitators and that it felt safe to share the facilitating role with a trained colleague, thus not being alone. Participants said:

Then I could really feel this way that “this is what we do together”. It depends as much on us as it depends on me if it goes well, and then I could kind of relax, and I think that also made the group relax better.

And that it is, precisely in the role of facilitator, that it feels like it has been good that there have actually been three of us,… that we ourselves have become secure in the role, because you know that there is a kind of lifeline, if you lose the thread, that you can always kind of reflect with the others and so on.

### Undertaking the Facilitator Role

Undertaking the facilitator role is another strategy for carrying the facilitator responsibility and involves striving to find a neutral role in the beginning of the ECR round. One way of doing that is to start with the practical issues (such as deciding on timeframes and confidentiality). It is also important to step out of the role as a HCP and one way to make this easier is to facilitate patient-cases that the facilitators are not clinically involved in, as exemplified by the following quote:

I have been involved in facilitating a patient [case] whom I have not met at all. And it was much easier for me, then I felt very neutral. I think it was much easier to be neutral when not knowing the patient. Then you had no opinion from the beginning.

Furthermore, undertaking the facilitator role includes standing back and balancing guiding. Standing back includes not giving own arguments and perspectives on the case but rather focusing on the process and fostering the dialogue to help HCPs to formulate their perspectives. Balancing guiding includes considering steering or letting go, as exemplified by this quote:

…something that has been a little difficult when someone has kind of drifted away, to steer it back without that person feeling like they are not allowed to say what they want …should we break… try to steer…maybe this still needs to be said and then I have let it go.

Moreover, an example of letting go included to let medical facts take time as it resulted in increased understanding of the difficult ethical situation.

Undertaking the facilitator role was described as thinking in circles and alternative approaches as well as trying to avoid being solving-oriented but rather tolerating the uncertainty. “…we as healthcare professionals tend to end up in this solution-focused way…we should try not to solve the problem when we…look at values ​​and norms and so on…”.

Lastly, to undertake the role involved to stay after the ECR round to get feedback to learn how to develop and improve the role. In our study, several facilitators described feeling satisfied with undertaking the facilitator role when the discussion turned out as productive and they expressed that they felt that they had assisted the participants in the ECR round to express their views. Expectations of their role were described as initially low, and several participants felt nervous and doubted their own performance. However, positive feelings were many times experienced after the ECR round.

## Discussion

The experiences of HCPs, under training to become facilitators, have been explored in Nordic paediatric oncology. Facilitator trainees’ main concern was delivering a meaningful experience of ethics support and fostering ethically good care for children. *Carrying the facilitator responsibility* is the core category and includes feeling and taking on the responsibility related to the moral case at hand in ECR, the ECR participants and their deliberation process. The great responsibility reinforces their perceived need for *achieved facilitator confidence*. Strategies for carrying the facilitator responsibility includes *allying* and *undertaking the facilitator role.*

*Carrying facilitator responsibility* emerged strongly from the data of the facilitator trainees. This resonates well with what the authors observed during the training of the HCPs as ERC facilitators. Despite, the absence of legal mandate and responsibility for the treatment decisions with respect to the care for children in paediatric oncology (Bartholdson et al., 2014), facilitator trainees took a heavy burden of responsibility on their own shoulders. Responsibility is a central concept in healthcare, often perceived by professionals as both a moral imperative and a source of pressure (Brown & Savulescu, 2019). Previous research has shown that HCPs tend to internalize responsibility not only for clinical outcomes but also for the ethical quality of care, sometimes to the extent that it becomes burdensome (Brown & Savulescu, 2019). This is particularly relevant in the context of CES, where facilitators may feel personally accountable for the success of the ERC process and its impact on patient care. Thus, this is important for the future training of ECR facilitators. Notably, the perceived responsibility in this study has impacted the Nordic training, emphasising more strongly shared responsibility, of the process and the outcome, in the group. An important factor is that this is in line with the ECR methodology (Molewijk et al., 2008). The success of an ECR cannot and should not solely be on the shoulders of the facilitators.

It is not surprising that achieved facilitator confidence was perceived as an important condition for carrying the facilitator responsibility, which was experienced as a heavy burden. Confidence could be connected to the need of ethical competencies of facilitators of ECR rounds; including knowledge, attitudes and skills (Molewijk et al., 2008). The knowledge could be compared to the perceived challenge of theoretical insufficiency, attitudes to the perceived responsibility of the case, and skills to the perceived responsibility of the process. Achieved facilitator confidence is not only a condition for performing as an ECR facilitator, it is also a goal of the training. Based on the results of the present study, the schedule has been changed to include more time for trainees to practice in the safe context of training to undertake the role by repeatedly facilitating real cases with trainee fellows. Somehow, it is understandable that the facilitator trainees in this study had not yet achieved it, since they were under training. There is an ongoing dialogue about core competencies of CES personnel, including facilitators. In the USA, core competencies have been developed for ethics consultants (Tarzian, 2013). In Europe, core competencies are still in the early phases of development and not yet formalized. One reason might be that CES is more pluralistic and that competencies might differ depending on the role of the CES personnel (for example, the facilitating role). Various authors who wrote about the competencies of CES personnel, have argued for non-theoretical competencies such as self-reflexivity, imagination and individual characteristics (Larcher et al., 2010; Slowther et al., 2004; Thornton, 2023). Moreover, another important competence of CES personnel in the European context is to strengthen the ethical competencies of all participants in CES i.e., HCPs (de Snoo-Trimp et al., 2020; Pergert et al., 2025). Furthermore, in a study with CES personnel, they perceived that if patients and families were to participate, CES personnel needed more confidence and experience in facilitating CES with only HCPs present (Billstein et al., 2025).

*Allying* emerged as a strategy for carrying the facilitator responsibility. This was expected since the trainees were encouraged to co-facilitate and collaborate when practicing in the clinic. To further encouraging allying, the teachers ensured that facilitator trainees practiced in couples, together with colleagues, from their own clinic during the facilitator course. This strategy turned out to be useful for carrying the facilitator responsibility and was also prominent when positioning ethics as facilitators were implementing ECR rounds in paediatric oncology (Pergert et al., 2024).

*Undertaking the facilitator role* entails adopting a facilitating rather than an authoritarian role, which has been claimed as most suitable in CES in Sweden (Pergert et al., 2025). Other than the facilitating role, a clear blueprint of what a good facilitator entails is not provided during the Nordic training. However, the training includes reflection about the role and meaning of being a good facilitator. Moreover, the training includes the tasks they need to do in ECR rounds, for example, foster a dialogue. The role of the facilitator can be undertaken by ethicists and chaplains as well as HCPs themselves. If ethicists have the opportunity to focus solely on CES they might assume a role as an expert. In our study participants were foremost HCPs, which is in line with CES personnel in Sweden who are most often HCPs rather than trained ethicists (Pergert et al., 2025). Thus, HCPs needed to step out of that role and step into the facilitator role. When HCPs step into the role as ethicists (Benedetti et al., 2023) and employ dual roles we believe they would benefit from using the strategy of undertaking the facilitator role.

### Strengths and Limitations

This study employed classic grounded theory to explore trainees’ experiences of facilitating ECR rounds in paediatric oncology. The methodology is suitable for uncovering underlying processes and generating middle-range theory. However, it could also be used to generate concepts and their properties (Glaser & Strauss, 1965, 1967), as in this study. In this paper, the conceptual categories remain closely tied to the study context and it thus quite descriptive. For instance, the use of “carrying the facilitator responsibility” rather than the more general term “Carrying the responsibility.” While “Carrying the facilitator responsibility” is grounded in the healthcare setting, the concept may also be applicable in other settings, though further theoretical sampling would be necessary to modify it (Glaser, 1978).

Although this study did not include observational data, which could be seen as a limitation, interviews are a well-established data source in classic grounded theory. The methodology is flexible regarding data types, as emphasized by the principle that “all is data” (Glaser & Strauss, 1967). However, the combination of focus group interviews and individual interviews provided rich, triangulated data. Another strength lies in the diversity of participants across all paediatric oncology centres in Sweden as well as focus group participants from all the Nordic countries, which supports the transferability of the results within similar healthcare contexts. However, the majority of participants came from Sweden, which may limit the transferability of the results across different cultural or institutional settings. The dual role of some researchers as both trainers and interviewers is a limitation as it may have introduced bias, potentially influencing participants’ responses in focus group interviews. However, this was partially mitigated by involving a non-teaching researcher conducting all individual interviews.

## Conclusion

In a context where HCPs are trained to become ECR facilitators, they take their role seriously. Facilitator trainees see it as their responsibility to deliver a meaningful experience of CES and to foster ethically good care for children when guiding ECR rounds in paediatric oncology. This perceived burden of responsibility should be addressed during facilitator training emphasising the use of the strategies allying, including shared responsibility, and undertaking the facilitator role. Training also needs to strengthen ethical competence to achieve facilitator confidence of the trainees. Further research is needed on what kind of core ethical competencies facilitators of ECR rounds need.

## Data Availability

Data will be made available upon reasonable request.

## References

[CR1] Bartholdson, C., Billstein, I., Molewijk, B., & Pergert, P. (2022). What outcomes of moral case deliberations are perceived important for healthcare professionals to handle moral challenges? A National cross-sectional study in paediatric oncology. *BMC Medical Ethics*, *23*(1), 108. 10.1186/s12910-022-00851-3 36368984 10.1186/s12910-022-00851-3 PMC9652809

[CR2] Bartholdson, C., Lützén, K., Blomgren, K., & Pergert, P. (2016). Clarifying perspectives: Ethics case reflection sessions in childhood cancer care. *Nursing Ethics*, *23*(4), 421–431. 10.1177/0969733015570511 25736273 10.1177/0969733015570511

[CR3] Bartholdson, C., Molewijk, B., Lutzen, K., Blomgren, K., & Pergert, P. (2018). Ethics case reflection sessions: Enablers and barriers. *Nursing Ethics*, *25*(2), 199–211. 10.1177/0969733017693471 29529973 10.1177/0969733017693471

[CR5] Benedetti, D. J., Marron, J. M., Thomas, S. M., Brown, A. E. C., Pyke-Grimm, K. A., Johnson, L. M., Unguru, Y., & Kodish, E. (2023). The role of ethicists in pediatric hematology/oncology: Current status and future needs. *Pediatric Blood and Cancer*, *70*(2), e30132. 10.1002/pbc.30132 36495529 10.1002/pbc.30132

[CR4] Bartholdson, C., Pergert, P., & Helgesson, G. (2014). Procedures for clinical ethics case reflections: An example from childhood cancer care. *Clinical Ethics*, *9*(2–3), 87–95.

[CR6] Billstein, I., Bartholdson, C., Castor, A., Molewijk, B., & Pergert, P. (2025). Ethics support personnel’s perceptions of patient and parent participation in clinical ethics support services in pediatric oncology. *BMC Medical Ethics*, *26*(1), 104. 10.1186/s12910-025-01267-5 40684215 10.1186/s12910-025-01267-5 PMC12275289

[CR7] Bloor, M., Frankland, J., Thomas, M., & Robson, K. (2001). *Focus groups in social research*. SAGE.

[CR8] Brown, R. C. H., & Savulescu, J. (2019). Responsibility in healthcare across time and agents. *Journal of Medical Ethics*, *45*(10), 636–644. 10.1136/medethics-2019-105382 31221764 10.1136/medethics-2019-105382 PMC6855791

[CR9] de Snoo-Trimp, J. C., de Vet, H. C. W., Widdershoven, G. A. M., Molewijk, A. C., & Svantesson, M. (2020). Moral competence, moral teamwork and moral action - the European moral case deliberation outcomes (Euro-MCD) instrument 2.0 and its revision process. *BMC Medical Ethics*, *21*(1), 53. 10.1186/s12910-020-00493-3 32616048 10.1186/s12910-020-00493-3 PMC7331166

[CR10] Delany, C., Feldman, S., Kameniar, B., & Gillam, L. (2024). Critical dialogue method of ethics consultation: Making clinical ethics facilitation visible and accessible. *Journal of Medical Ethics*, *51*(1), 10–16. 10.1136/jme-2024-109927 38977289 10.1136/jme-2024-109927

[CR11] Dörries, A., Simon, A., Vollmann, J., & Neitzke, G. (2014). The impact of an ethics training programme on the success of clinical ethics services. *Clinical Ethics*, *9*(1), 36–44.

[CR12] Forde, R., & Pedersen, R. (2011). Clinical ethics committees in norway: What do they do, and does it make a difference? *Cambridge Quarterly of Healthcare Ethics*, *20*(3), 389–395. 10.1017/s0963180111000077 21676326 10.1017/S0963180111000077

[CR13] Glaser, B. G. (1978). *Theoretical sensitivity: Advances in the methodology of grounded theory*. Sociology Press.

[CR14] Glaser, B. G. (1998). *Doing grounded theory: Issues and discussions*. Sociology Press.

[CR15] Glaser, B. G., & Strauss, A. L. (1965). *Awareness of dying* (11th pr., 1st ed.). Aldine Publishing Company.

[CR16] ______ . (1967). *The discovery of grounded theory: Strategies for qualitative research*. Aldine.

[CR17] Haan, M. M., van Gurp, J. L. P., Naber, S. M., & Groenewoud, A. S. (2018). Impact of moral case deliberation in healthcare settings: A literature review. *BMC Medical Ethics*, *19*(1), 85. 10.1186/s12910-018-0325-y 30400913 10.1186/s12910-018-0325-y PMC6219174

[CR18] Hem, M. H., Pedersen, R., Norvoll, R., & Molewijk, B. (2015). Evaluating clinical ethics support in mental healthcare: A systematic literature review. *Nursing Ethics*, *22*(4), 452–466. 10.1177/0969733014539783 25091004 10.1177/0969733014539783

[CR19] Janssens, R. M., van Zadelhoff, E., van Loo, G., Widdershoven, G., & Molewijk, B. A. (2015). Evaluation and perceived results of moral case deliberation: A mixed methods study. *Nursing Ethics*, *22*(8), 870–880. 10.1177/0969733014557115 25542405 10.1177/0969733014557115

[CR20] Larcher, V., Slowther, A. M., & Watson, A. R. (2010). Core competencies for clinical ethics committees. *Clinical Medicine*, *10*(1), 30–33. 10.7861/clinmedicine.10-1-30 20408302 10.7861/clinmedicine.10-1-30 PMC4954474

[CR21] Metselaar, S., Molewijk, B., & Widdershoven, G. (2015). Beyond recommendation and mediation: Moral case deliberation as Moral learning in dialogue. *The American Journal of Bioethics*, *15*(1), 50–51. 10.1080/15265161.2014.975381 10.1080/15265161.2014.975381 25562230

[CR22] Molewijk, B., Abma, T., Stolper, M., & Widdershoven, G. (2008). Teaching ethics in the clinic. The theory and practice of moral case deliberation. *Journal of Medical Ethics*, *34*(2), 120–124. 10.1136/jme.2006.018580 18234952 10.1136/jme.2006.018580

[CR23] Pergert, P., Molewijk, B., & Bartholdson, C. (2024). Positioning ethics when direct patient care is prioritized: Experiences from implementing ethics case reflection rounds in childhood cancer care. *H E C Forum, **37*(3), 345–356. 10.1007/s10730-024-09541-6 10.1007/s10730-024-09541-6 PMC12313733 39487914

[CR24] Pergert, P., Svantesson, M., Bartholdson, C., Bremer, A., Brännström, M., Grönlund, F., Juth, C., N., & Björk, J. (2025). Case-based clinical ethics support—a description and normative discussion of methodological issues from the Swedish perspective. *H E C Forum, *Accessed November 19, 2025, from https://link.springer.com/article/10.1007/s10730-025-09566-5 10.1007/s10730-025-09566-5 10.1007/s10730-025-09566-5 PMC13194189 41076596

[CR25] Plantinga, M., Molewijk, B., de Bree, M., Moraal, M., Verkerk, M., & Widdershoven, G. A. (2012). Training healthcare professionals as moral case deliberation facilitators: Evaluation of a Dutch training programme. *Journal of Medical Ethics*, *38*(10), 630–635. 10.1136/medethics-2012-100546 22844030 10.1136/medethics-2012-100546

[CR26] Silén, M., & Svantesson, M. (2019). Impact of clinical ethics support on daily practice-First-line managers’ experiences in the Euro-MCD project. *Journal of Nursing Management*, *27*(7), 1374–1383. 10.1111/jonm.12818 31220384 10.1111/jonm.12818

[CR27] Slowther, A., Johnston, C., Goodall, J., & Hope, T. (2004). Development of clinical ethics committees. *The BMJ*, *328*(7445), 950–952. 10.1136/bmj.328.7445.950 15087349 10.1136/bmj.328.7445.950 PMC390221

[CR28] Stolper, M. (2016). *Learning by doing: Developing moral case deliberation in health care*. Vrije Universiteit Amsterdam.

[CR29] Stolper, M., Molewijk, B., & Widdershoven, G. (2015). Learning by doing. Training health care professionals to become facilitator of moral case deliberation. *H EC Forum*, *27*(1), 47–59. 10.1007/s10730-014-9251-7 10.1007/s10730-014-9251-7 25218568

[CR30] Stolper, M., Molewijk, B., & Widdershoven, G. (2016). Bioethics education in clinical settings: Theory and practice of the dilemma method of moral case deliberation. *BMC Medical Ethics*, *17*(1), 1. 27448597 10.1186/s12910-016-0125-1 PMC4957934

[CR31] Tarzian, A. J. (2013). Health care ethics consultation: An update on core competencies and emerging standards from the American society for bioethics and humanities’ core competencies update task force. *American Journal of Bioethics*, *13*(2), 3–13. 10.1080/15265161.2012.750388 10.1080/15265161.2012.750388 23391049

[CR32] Thornton, A. (2023). *Facing the complexity gap: Developing leaders’ reasoning skills to meet the complex task demands of their roles* . PhD Diss. The University of Western Australia.

[CR33] Weiner, C., Pergert, P., Molewijk, B., Castor, A., & Bartholdson, C. (2021). Perceptions of important outcomes of moral case deliberations: A qualitative study among healthcare professionals in childhood cancer care. *BMC Medical Ethics*, *22*(1), 27. 10.1186/s12910-021-00597-4 33731101 10.1186/s12910-021-00597-4 PMC7970765

